# Cisplatin-Induced Anorexia and Pica Behavior in Rats Enhanced by Chronic Stress Pretreatment

**DOI:** 10.3389/fphar.2022.913124

**Published:** 2022-07-15

**Authors:** Zhijun Guo, Jingjing Duan, Yitian Chen, Weijia Cai, Chenghua Yang, Zhen Yang, Xiufeng Liu, Feng Xu

**Affiliations:** ^1^ School of Pharmaceutical Sciences, Southern Medical University, Guangzhou, China; ^2^ Fengxian Hospital, Southern Medical University, Shanghai, China

**Keywords:** chronic unpredictable mild stress, anorexia, pica, cisplatin, chemotherapy-induced nausea and vomiting

## Abstract

**Background:** Chemotherapy-induced nausea and vomiting severely impairs the treatment and prognosis of cancer patients. Depressive mood disorder might aggravate nausea and vomiting in cancer patients; however, the role of neurotransmitters and receptors involved in the mediation of emesis and nausea is still not well elaborated.

**Methods:** The study was carried out based on the chronic unpredictable mild stress–induced depression-like phenotype rat model and cisplatin-induced pica rat model establishment. Forty male Sprague–Dawley rats were randomized into the non-treated control group and the chronic stress group, which were exposed to 8 weeks of stress. Each group was then sub-divided into vehicle subgroups (*n* = 10) and cisplatin subgroups (*n* = 10) which were given cisplatin to induce pica behavior. Kaolin and food intake were recorded after administration. The medulla oblongata and ileum tissues were obtained. Neurotransmitters involved in the mediation of emesis and nausea (5-HT, DA, SP, and AEA) were detected using an ELISA kit. Vomit-related receptors (5-HT_3_R, DA_2_R, NK_1_R, and CB_1_R) in tissues were assayed for mRNA and protein expression by RT-qPCR and Western blotting.

**Results:** Behavioral test and sucrose preference validated that depression-like phenotype rat models were established successfully. The kaolin consumption test confirmed that chronic stress pretreatment aggravated anorexia and pica behavior. Vomiting-related molecules’ data showed that chronic stress exposure increased 5-HT and SP levels in the medulla oblongata. Vomiting-related receptor expression data showed that chronic stress pretreatment upregulated 5-HT_3_R, DA_2_R, and NK_1_R expressions and downregulated the CB_1_R expression in the medulla oblongata. However, chronic stress pretreatment downregulated 5-HT_3_R, DA_2_R, and NK_1_R expressions and upregulated the CB_1_R expression in the ileum.

**Conclusion:** Chronic stress pretreatment aggravates anorexia and vomiting progress, which might be *via* altering neurotransmitters and receptors involved in the mediation of emesis and the nausea level and expression in the central nervous system.

## Introduction

Chemotherapy-induced nausea and vomiting (CINV) is a critically adverse drug reaction in cancer patients (Aapro, 2018), which severely disturbs treatment decision and impairs overall quality of life and therapeutic outcome (Hawkins and Grunberg, 2009; Bray et al., 2018; Natale, 2018).

Serotonin (5-HT) and substance P (SP) are key factors involved in the vomiting process (Darmani and Ray, 2009). When 5-HT or/and SP combines with the corresponding receptors, the vomiting center in the medulla oblongata is triggered and thus results in vomiting. Although new therapeutics have been developed for nausea and vomiting control, CINV is still very challenging for cancer chemotherapy patients (Jordan et al., 2015; Warr, 2018). Up to now, CINV has remained a thorny issue in cancer chemotherapy (Dranitsaris et al., 2017). Approximately 9–30% of cancer patients still suffer from serious vomiting despite current anti-emetic medications (Mersiades et al., 2018). A further complication is that many risk factors are identified to aggravate CINV, including history of nausea/vomiting, expectancy of CINV, alcohol intake, and anxiety/depression (Mosa et al., 2020).

In our previous work, we found that the frequency and extent of subjectively experienced adverse drug reactions (anorexia, nausea, and fatigue) in cancer chemotherapy patients seemed to be well in line with the severity of their depression (Zhou et al., 2010). Chronic stress pretreatment might aggravate the vomiting process; however, the underlying mechanism is still not clear. In addition, many documents indicated that the gastrointestinal function was affected by a specific psychological mood disorder status such as depression and anxiety (Peng and Liang, 1999; Liu et al., 2015), further indicating that chronic stress pretreatment is related to changes in the gastrointestinal function.

In this study, we hypothesized that chronic stress pretreatment might aggravate nausea and vomiting. Due to the special physiological and anatomical structure, rats are lacking a vomiting response. Non-nutritive substance consumption, such as kaolin consumption (pica behavior), can indirectly reflect the degree of vomiting in rats (Borner et al., 2020). We examined the kaolin consumption in this study to explore the vomiting response in rats. As the vomiting process involves many molecules and receptors (Li and Xu, 2020), this study focused on neurotransmitters involved in the mediation of emesis and nausea 5-HT, DA, SP and AEA level, 5-HT_3_R, DA_2_R, NK_1_R, and CB_1_R expressions in the central nervous system and aimed to explore the relevant molecule profile of chronic stress aggravating CINV based on chronic unpredictable mild stress (CUMS) and/or the pica rat model.

## Materials and Methods

### Animals

Male Sprague–Dawley rats weighing 120–150 g were purchased from Shanghai Jiesijie Laboratory Animal Technology Co., Ltd. (Animal Quality Certificate: 20180004058940). Rats were housed in a specific pathogen-free (SPF) laboratory with a regular 12-h light (06:00–18:00)-dark (18:00–06:00) cycle, at the Laboratory Animal Center, East China Normal University, Shanghai. All rats were allowed to adapt to the new environment for 1 week before CUMS model establishment. The body weight of rats was measured biweekly. Animal procedures complied with the ARRIVE guidelines. This study was reviewed and approved by the Animal Research Ethics Committee in Fengxian Hospital, Southern Medical University (No. 201920143).

### Behavioral Testing—Open Field Test

The OFT was used to evaluate the locomotion activity and exploratory behavior of rats. Each rat was individually placed at the center of a box (100 cm × 100 cm × 40 cm) divided into 25 cm × 25 cm^2^ squares and observed for 5 min. The crossing numbers of squares (locomotion activity) and the rearing times (exploratory behavior) were monitored using a ZSZFT Video Analysis System (ZSZRDC Science and Technology Co., Ltd., China). The OFT was only conducted before and after 8 weeks of modeling to maintain a relatively unfamiliar environment for rats.

### Sucrose Preference Test

The SPT was used to detect the anhedonia status in depression-like phenotype rats (Liu et al., 2018). Rats were individually housed in cages for isolation adaption. Sucrose training for 48 h helped the rats adapt to the sucrose solution. At the first 24 h, two bottles of 1% sucrose solution (Sigma, St. Louis, MO, United States) were given to the rats. At the second 24 h, a bottle of water and a bottle of 1% sucrose solution were given to the rats. After 23 h of deprivation of water and food, a bottle of water and a bottle of 1% sucrose solution were given to the rats, and the intake of water and sucrose solution for 1 h was recorded. The positions of the two bottles were exchanged every 6 h to eliminate unilateral preference during the whole test.

Sucrose preference%=(sucrose solution consumption/(sucrose solution+water consumption))×100%.

### Chronic Stress Model

After OFT and SPT primary screening, rats with normal behavior were divided into the non-treated control group (*n* = 20) and the chronic stress group (*n* = 20) with a random number table. Rats in the chronic stress group were individually housed in cages and exposed to the following stressors in a random order once a day for 8 weeks: restraint (activity restriction in a 30 cm × 6 cm plastic bottle, 1 h), hot water swimming (45°C, 5 min), cold water swimming (4°C, 5 min), clip tail (clip 1 cm from the end of the tail, 1 min), cage tilting (45°, 24 h), horizontal shaking (30 min), damp padding (24 h), noise interference (industrial noise from a media player, 2 h), and day/night inversion (24 h) (Antoniuk et al., 2019; Li et al., 2019; Zhao et al., 2020). The use of the same stress for two consecutive days was avoided for the purpose of unpredictability. Rats in the non-treated control group were grouped in cages and fed normally without any stress during this period.

### Cisplatin-Induced Pica Model

Here, 2% Arabic gum solution was prepared by stirring Arabic gum powder (Shanghai Macklin Biochemical Co., Ltd.) in distilled water at room temperature until it was completely dissolved. Then, 500 g kaolin (Shanghai Yuanye Biological Technology Co., Ltd.) and 200 ml of 2% Arabic gum solution were mixed to make a thick paste. The paste was dried at room temperature and molded into chow food in the shape of pellets. After 8 weeks of chronic stress, rats in both the non-treated control group and the chronic stress group were further sub-divided into the vehicle subgroup (normal saline) and the cisplatin subgroup (cisplatin administration), respectively, as follows: non-treated/saline group (*n* = 10), non-treated/cisplatin group (*n* = 10), CUMS/cisplatin group (*n* = 10), and CUMS group (*n* = 10). Rats in the non-treated/saline group, non-treated/cisplatin group, and CUMS/cisplatin group were individually housed in cages and given kaolin pellets for 1 week of adaptation. Food was provided simultaneously during the cisplatin-induced pica model. Non-treated/cisplatin group and CUMS/cisplatin group rats were then given 6 mg/kg cisplatin (Qilu Pharmaceutical Co., Ltd.) intraperitoneally; meanwhile, rats in the non-treated/saline group were given normal saline intraperitoneally. The intake of kaolin and food was recorded at 24 h after administration. For the cisplatin-induced pica behavior model, rats were not given any stress to avoid affecting kaolin and food intake.

### Measurement of Neurotransmitters and Receptors Involved in the Mediation of Emesis and Nausea

To further investigate the effect of chronic stress pretreatment on vomit-related molecules, we examined the vomit-related neurotransmitters’ levels and vomit-related receptors’ expression in the non-treated/saline group and CUMS group. Then, 24 h after cisplatin administration, the medulla oblongata and ileum tissues of rats in the non-treated/saline group and CUMS group were collected. Portions of tissues were homogenized at 4°C. The levels of 5-HT, DA, SP, and AEA in the tissue homogenate were detected using the ELISA kit (Jiangsu Jianglai Biotechnology Co., Ltd.), according to the manufacturer’s protocol. Portions of tissues were preserved in liquid nitrogen for receptor mRNA and protein expression measurement.

For RT-qPCR measurement, briefly, total RNA of the medulla oblongata and ileum tissues were extracted with TRIzol (Invitrogen™, Carlsbad, United States), according to the protocol, and the RNA concentration was measured using a NanoDrop ND-100 spectrophotometer (Thermo Scientific, Wilmington, DE, United States). Tli RNaseH Plus was used according to the manufacturer’s protocol. The 2^−ΔΔCT^ method was used to normalize the fold change in the gene expression. The primer sequences are as follows:

*GADPH* forward primer, 5′-CAA​GAA​GGT​GGT​GAA​GCA​G-3′ and reverse primer, 5′-CAA​AGG​TGG​AAG​AAT​GGG-3’; *5-HT*_*3*_ *R* forward primer, 5′-CTG​TCC​TCC​ATC​CGC​CAC​TCC-3′ and reverse primer, 5′- CAG​CAG​CCT​GTC​CAG​CAC​ATA​TC-3’; *DA*_*2*_ *R* forward primer, 5′- CAC​ACG​CTA​CAG​CTC​CAA​G-3′ and reverse primer, 5′- GAA​GGA​CAG​GAC​CCA​GAC​A-3’; *NK*_*1*_ *R* forward primer, 5′- CCG​CTA​CCA​TGA​GCA​AGT-3′ and reverse primer, 5′- AGG​GCA​GGA​GGA​AGA​AGA-3’; and *CB*_*1*_ *R* forward primer, 5′- GGA​CCC​AGA​AGA​GCA​TCA-3′ and reverse primer, 5′- ATC​AAC​ACC​ACC​AGG​ATC​A-3’. The gene expression was normalized with the following: experimental group: target gene/housekeeping gene; control group: target gene/housekeeping gene).

For Western blot, briefly, the medulla oblongata and ileum tissues were individually ground and sonicated in RIPA buffer (Beyotime, China) for 30 min and centrifuged at 14,000 g for 20 min at 4°C. The supernatant was quantified using the BCA protein assay kit (Beyotime, China), analyzed by 10% SDS-PAGE (Beyotime, China), and transferred onto a nitrocellulose filter membrane (Pall, America). After 1 h of blocking, the membranes were incubated with the primary antibody (5-HT_3_R antibody, CB_1_R antibody, and β-actin antibody were purchased from Proteintech, NK_1_R antibody was purchased from Immunoway, and DA_2_R antibody was purchased from Bioss; dilution rate: 5-HT_3_R, CB_1_R, NK_1_R, and DA_2_R were 1:1000, and β-actin was 1:5000) overnight at 4°C, followed by incubation with secondary antibodies (Goat anti-rabbit antibody and goat anti-mouse antibody were purchased from Proteintech; dilution rate: goat anti-rabbit and goat anti-mouse were 1:5000), and detected with an ECL buffer (NCM Biotech, China). The intensity of each band was determined by ImageJ software. The protein expression was normalized with the following: experimental group: target protein/housekeeping protein; control group: target protein/housekeeping protein).

### Statistical Analysis

All analysis was performed using SPSS 19.0. Data were expressed as mean ± SD. Statistical comparisons between the two groups were performed using the two independent sample t-tests, and the same group was performed with paired samples’ t-test. The inter-groups were performed using one-way analysis of variance (ANOVA). Repeated measurement data were performed using two-way repeated measures ANOVA. LSD and Dunnett’s T3 were used for *post hoc* multiple comparisons. *p* < 0.05 was considered to be statistically significant.

## Results

### Chronic Stress Model Validation

**FIGURE 1:**
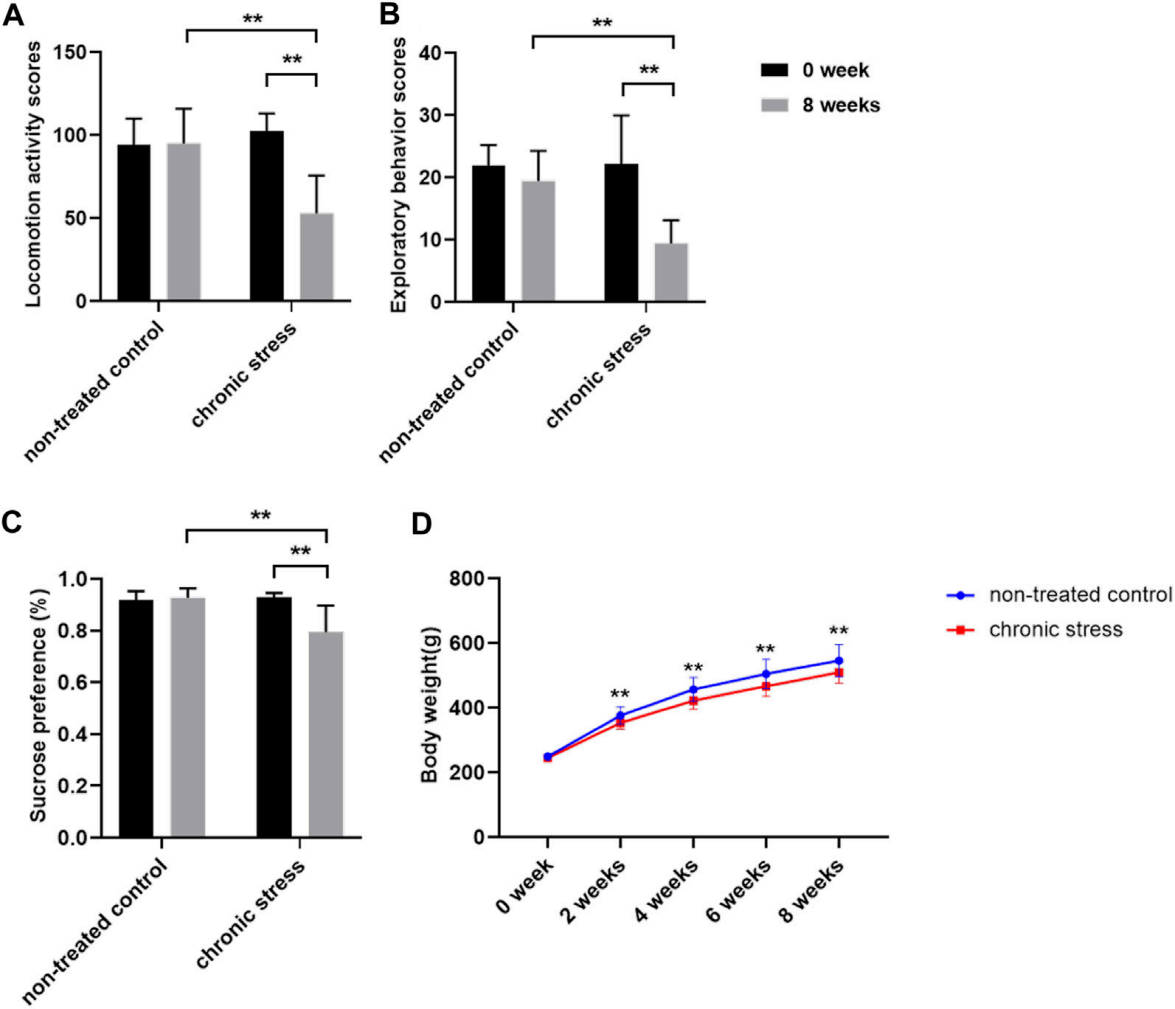
Chronic stress model was established successfully (*n* = 20/group). **(A)** Locomotion activity scores. **(B)** Exploratory behavior scores. **(C)** Sucrose preference. **(D)** Body weight. Data were expressed as mean ± SD. **p < 0.01 (statistical comparisons between the two groups were performed using the two independent sample t-test, and the same group was performed with the paired sample t-test. Repeated measurement data was performed using two-way repeated measures ANOVA.).

After 8 weeks of CUMS model establishment, the locomotion activity scores, exploratory behavior scores, and sucrose preference were decreased in chronic stress group rats compared to baseline (*p* < 0.01). However, there was little change in the non-treated control rats over time (Figures 1A–C). The results showed that depression-like behavior was successfully induced in rats in the chronic stress group compared with rats in the non-treated control group. Compared to the non-treated control group, a moderate reduction in the body weight in chronic stress group rats was observed from the second week of CUMS model establishment (Figure 1D). Behavioral test and sucrose preference test results indicated that the chronic stress–induced depression-like phenotype rat model was successfully established.

### Chronic Stress Pretreatment Aggravates Cisplatin-Induced Anorexia and Pica Behavior

**FIGURE 2:**
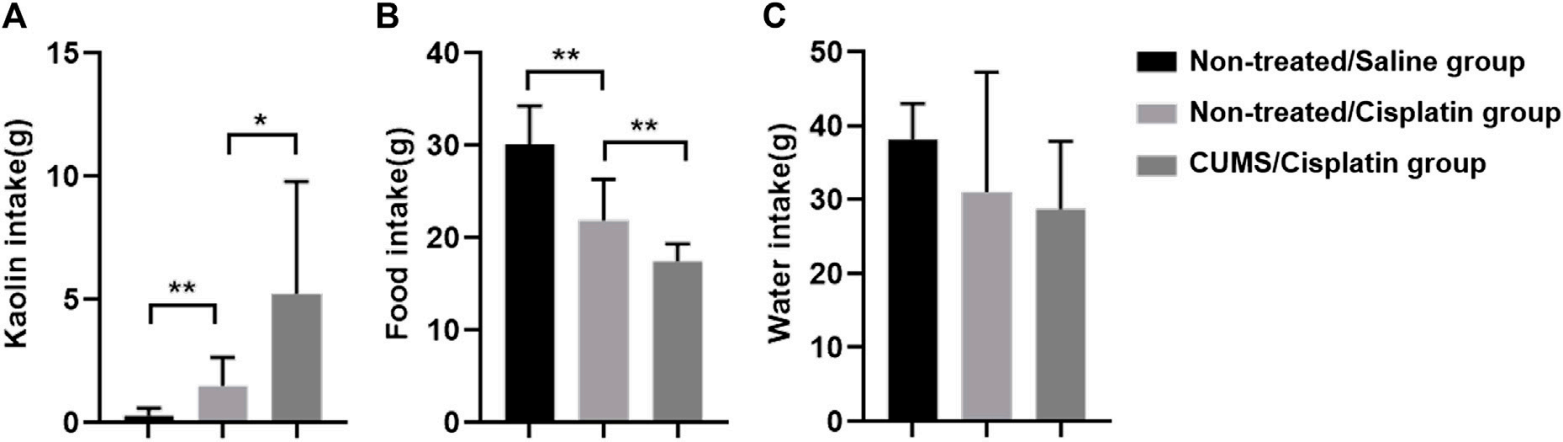
Chronic stress pretreatment enhanced cisplatin (6 mg/kg)-induced anorexia and pica behavior in rats (*n* = 10/group). **(A)** Kaolin intake. **(B)** Food intake. **(C)** Water intake. *p < 0.05 and **p < 0.01(statistical comparisons in the inter-groups were performed using one-way analysis of variance (ANOVA).).

Kaolin pellets were given to rats in the non-treated/saline group, non-treated/cisplatin group, and CUMS/cisplatin group for 1 week of adaptation. 24 h after administration, kaolin consumption was significantly increased both in the non-treated/cisplatin group and CUMS/cisplatin group compared to that in the non-treated/saline group (Figure 2A). Furthermore, the kaolin consumption in the CUMS/cisplatin group increased more significantly than that in the non-treated/cisplatin group (*p* < 0.01), suggesting that chronic stress aggravated vomit-like behavior (increased kaolin consumption). In addition, food intake was decreased in the non-treated/cisplatin group and CUMS/cisplatin group (Figure 2B). Chronic stress pretreatment enhanced the reduction of food in the CUMS/cisplatin group. However, there was no significant change in water consumption among the three groups (Figure 2C). Data revealed that cisplatin administration increased the kaolin consumption, and chronic stress pretreatment enhanced this effect in rats.

### Chronic Stress Increases 5-HT and SP Levels, Upregulates 5-HT_3_R, DA_2_R, and NK_1_R, and Downregulates the CB_1_R Expression in the Medulla Oblongata

**FIGURE 3:**
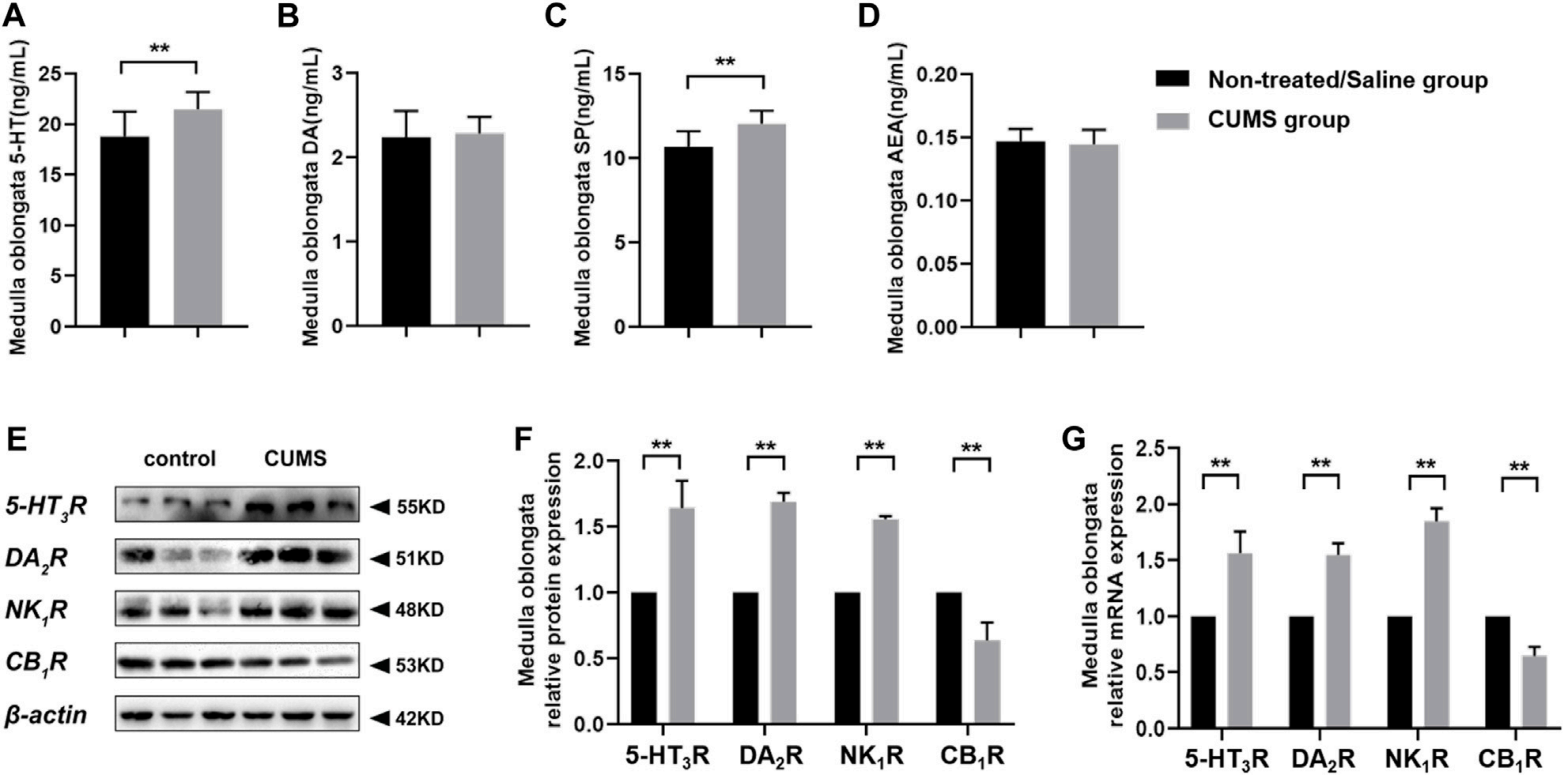
Chronic stress increased 5-HT and SP levels, upregulated 5-HT_3_R, DA_2_R, and NK_1_R expressions, and downregulated the CB_1_R expression in the CNS (*n* = 10/group). **(A–D)** Vomit-related neurotransmitter levels in the medulla oblongata. **(E)** Representative Western blotting bands of vomit-related receptors. **(F)** Relative protein expression of vomit-related receptors in the medulla oblongata (normalization: experimental group (target protein/housekeeping protein)/control group (target protein/housekeeping protein)). **(G)** Relative mRNA expression of vomit-related receptors in the medulla oblongata (normalization: experimental group (target gene/housekeeping gene)/control group (target gene/housekeeping gene)). Data were expressed as mean ± SD. **p < 0.01 (statistical comparisons between the two groups were performed using the two independent sample t-test).

We further investigated the neurotransmitters and receptors involved in the mediation of emesis and the nausea level and receptor expression in the central nervous system (CNS) of the non-treated/saline group and CUMS group. Compared with the non-treated/saline group, chronic stress significantly increased 5-HT and SP levels in the CUMS group (*p* < 0.01). However, little change occurred in DA and AEA levels between the non-treated/saline group and CUMS group (Figures 3A–D). The immunoblotting and RT-qPCR assay results showed that chronic stress obviously upregulated 5-HT_3_R, DA_2_R, and NK_1_R expressions but downregulated the CB_1_R expression in the medulla oblongata in the CUMS group (*p* < 0.01) compared to those in the non-treated/saline group (Figures 3E–G). Results showed that chronic stress increased the levels of vomit-related neurotransmitters 5-HT and SP and altered vomit-related receptors’ expression in the medulla oblongata, which means that more vomit-related neurotransmitters could combine with the corresponding vomit-related receptors and then affect the vomiting process.

### Chronic Stress Does Not Alter 5-HT, DA, SP, and AEA Levels, but Downregulates 5-HT_3_R, DA_2_R, and NK_1_R Expressions, and Upregulates the CB_1_R Expression in Ileum Tissue

**FIGURE 4:**
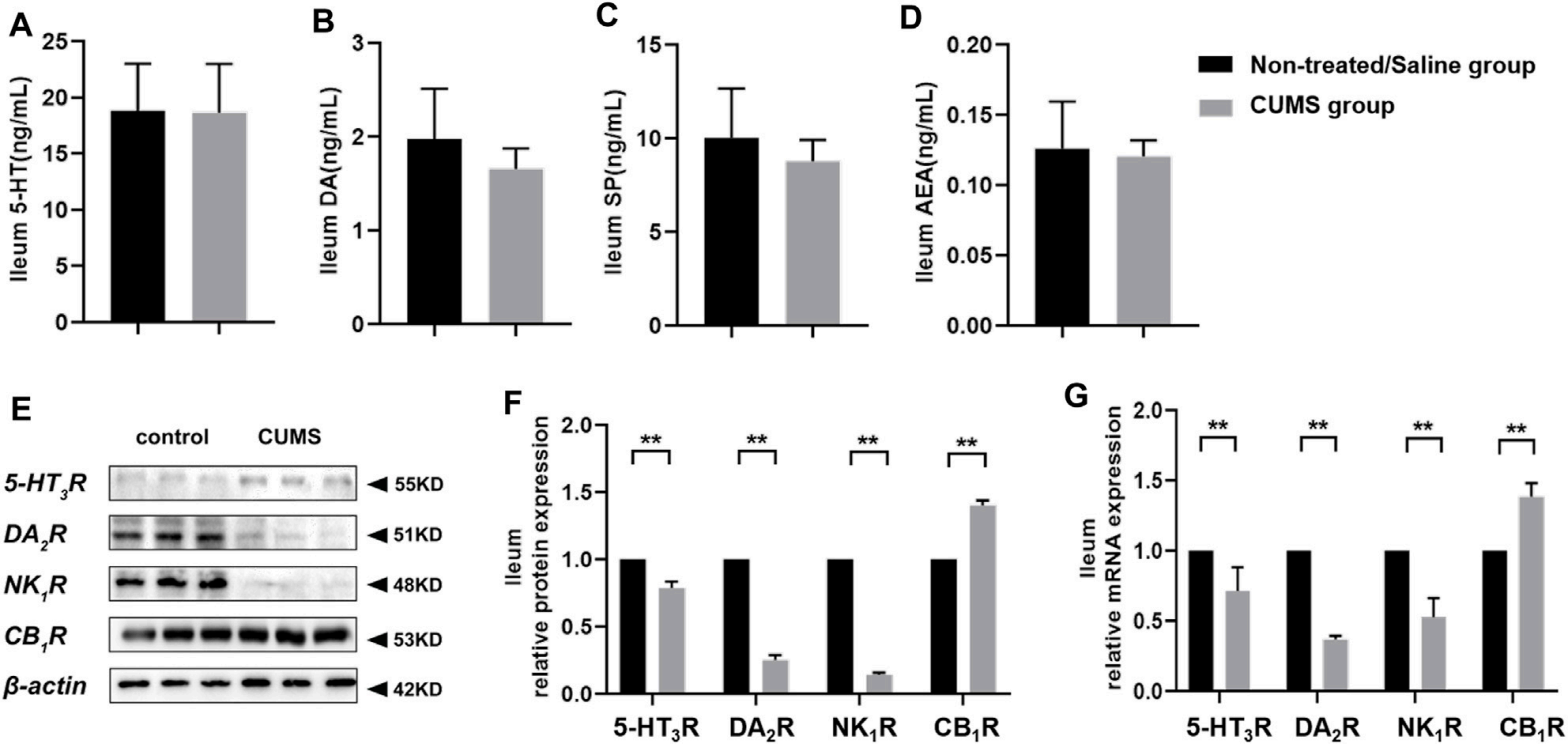
Chronic stress did not alter 5-HT, DA, SP, and AEA levels, downregulated 5-HT_3_R, DA_2_R, and NK_1_R expressions, and upregulated the CB_1_R expression in the peripheral system (*n* = 10/group). **(A–D)** Vomit-related neurotransmitter levels in the ileum. **(E,F)** Representative Western blotting bands and relative protein expressions of vomit-related receptors in the ileum [normalization: experimental group (target protein/housekeeping protein)/control group (target protein/housekeeping protein)]. **(G)** Relative mRNA expression of vomit-related receptors in the ileum [normalization: experimental group (target gene/housekeeping gene)/control group (target gene/housekeeping gene)]. Data were expressed as mean ± SD. **p < 0.01 (statistical comparisons between the two groups were performed using the two independent sample t-test).

To figure out the effect of chronic stress on neurotransmitters involved in the mediation of emesis and nausea in ileum tissue, we measured the 5-HT, DA, SP, and AEA levels and 5-HT_3_R, DA_2_R, NK_1_R, and CB_1_R expressions. ELISA assay revealed that there was no significant change for relevant molecule levels between the non-treated/saline group and CUMS group (Figures 4A–D). In addition, the Western blot assay and RT-qPCR results displayed that chronic stress downregulated 5-HT_3_R, DA_2_R, and NK_1_R expressions and upregulated the CB_1_R expression in the ileum in the CUMS group (*p* < 0.01) compared to the non-treated/saline group (Figures 4E–G). We noticed that chronic stress did not significantly change the vomit-related neurotransmitters’ level in the ileum, and the alteration of vomit-related receptors in the ileum was inconsistent with that in the CNS.

## Discussion

In this study, we established a chronic stress–induced depression-like behavior and cisplatin–induced pica rat model. Chronic stress pretreatment decreases food intake and significantly increases kaolin consumption after cisplatin injection, which suggest that chronic stress pretreatment induces anorexia and aggravates cisplatin-induced pica behavior. We explored the relevant molecule profile in this process and found for the first time that chronic stress might aggravate the vomiting progress *via* altering the level of neurotransmitters and the expression of receptors involved in the mediation of emesis and nausea in the central nervous system of rats.

Clinical evidences indicated that anorexia nervosa often occurs in individuals with stress, anxiety-like, or depression-like behavior (Lian et al., 2017; Lloyd et al., 2019), suggesting the possible association between mood disorder and anorexia. Similar to this finding, our previous work also noted that cancer chemotherapy patients with severe depression seemed more likely to have frequent and severe adverse drug reactions including anorexia, nausea, and fatigue (Zhou et al., 2010). Nausea and vomiting, one of the most terrible adverse reactions experienced by cancer patients, is associated with multiple risk factors including gender, age, anticancer drug dose, history of morning sickness, and alcohol intake. More and more researchers believe that depression and the anxiety mood disorder play a great role in chemotherapy-induced nausea and vomiting (Navari and Aapro, 2016; Kawazoe et al., 2018; Tsuji et al., 2019; Guo and Xu, 2020; Nasu et al., 2020). In addition, expectancy psychology of nausea and vomiting promote vomiting progress for cancer patients (Mosa et al., 2020).

Due to the special physiological structure, rodent animals like rats lack vomiting reflex. Therefore, vomiting in rats is assessed by pica behavior (Takeda et al., 1993). Cisplatin is a classic anticancer drug with high emetogenicity (Bamias et al., 2019), which causes severe nausea and vomiting, especially acute vomiting in cancer patients (Zhang et al., 2018). The cisplatin-induced pica rat model is a widely used animal model for antiemetic screening. Based on this pica model, we further confirmed that chronic stress–induced depression-like phenotype rats are more prone to displaying pica behavior (vomit-like behavior).

As the nausea and vomiting process involves various neurotransmitters and receptors (Di Maio et al., 2018), we studied the 5-HT, DA, SP, and AEA levels and 5-HT_3_R, DA_2_R, NK_1_R, and CB_1_R expressions in tissue and explored their possible change during the chronic stress process. The results suggested that 5-HT and SP elevation, as 5-HT_3_R and NK_1_R expressions increase in the CNS, might play a crucial role in the chronic stress aggravating vomit. As elevated levels of 5-HT and SP are bound with highly expressed 5-HT_3_R/NK_1_R, vomiting response is triggered easily, frequently, and severely (Phillips et al., 2016). Why there is no change in 5-HT and SP levels in the ileum is worthy of further in-depth research.

In this study, we mainly focused on the effect of chronic stress on vomit-related molecules’ level while not considering the antiemetic drug efficacy of rats. It would be beneficial to investigate the efficacy for the cisplatin-induced pica behavior in our chronic stress experimental condition in rats in future. In addition, recently, data have shown that different bedding in the cages may influence food intake (Lovasz et al., 2020); bedding change in our CUMS model establishment process might interfere with the results, and it might be an important limitation to the present study.

## Conclusion

In this study, chronic stress aggravates cisplatin-induced anorexia and vomiting progress in rats which might be increasing the level of neurotransmitters 5-HT and SP, upregulating the expressions of receptors 5-HT_3_R, DA_2_R, and NK_1_R, and downregulating the CB_1_R expression in the central nervous system.

## Data Availability

The raw data supporting the conclusions of this article will be made available by the authors, without undue reservation.
